# Effect of Unilateral Shoulder Disorder on the Stance Phase of Human Gait

**DOI:** 10.1155/2022/8205879

**Published:** 2022-04-25

**Authors:** Martin Missmann, Gerald Aron, Thomas Stöggl, Michael Pirchl, Vincent Grote, Michael J. Fischer

**Affiliations:** ^1^Ludwig Boltzmann Institute for Rehabilitation Research, Vienna, Austria; ^2^Austrian Workers' Compensation Board (AUVA), Innsbruck, Austria; ^3^VAMED Rehabilitation Center Kitzbuehel, Kitzbuehel, Austria; ^4^Piz-Therapie, Saalfelden, Austria; ^5^Department of Sport and Exercise Science, University of Salzburg, Salzburg, Austria; ^6^Hannover Medical School MHH, Clinic for Rehabilitation Medicine, Hannover, Germany

## Abstract

**Background:**

Gait analysis systems serve as important tools for assessing disturbed gait patterns. Amongst other factors, functional limitations of the shoulder joint may relate to such disturbances. Patient-reported outcome measures, assessment of pain, and active range of motion are commonly used to describe shoulder impairment.

**Purpose:**

The aim of this cohort study was to evaluate the impact of unilateral limitations of shoulder mobility and pain on gait patterns and to detect correlations between pain, shoulder mobility, and particular phases of human gait using a Zebris gait analysis system.

**Methods:**

20 subjects with unilaterally restricted mobility and pain of the affected shoulder and a control group of 10 healthy subjects underwent a gait analysis. Various gait parameters, the DASH score, pain at rest and movement of the affected shoulder, and the active range of motion (aROM) for shoulder flexion and abduction were recorded.

**Results:**

We determined significant differences of the duration of the loading response (*p* = 0.021), midstance (*p* = 0.033), and the terminal stance phase (*p* = 0.019) between the shoulder group and the control group, with a shorter loading response phase and a longer terminal stance phase of the affected side in the shoulder group. In the shoulder group, we found significant correlations between the DASH and the duration of the midstance phase (*p* = 0.023) and the terminal stance phase (*p* = 0.038). In addition, there was a significant correlation between shoulder flexion and the duration of the midstance phase (*p* = 0.047).

## 1. Background

Walking is a method of locomotion involving the use of the two legs alternately to provide both support and propulsion. Human gait evolved from quadruped locomotion with arm movements and stepping being controlled by spinal central pattern generators. While walking, the arms move rhythmically out of phase with the corresponding leg, which is caused by alternating activity of the muscles of the shoulder girdle and the upper limb [1], with highest coherence values between the deltoid and the proximal leg muscles [2]. Rhythmic swinging of the arms is a universal feature of human bipedal gait and is likely subject to a mix of cortical and lower-level neural control [3, 4].

Neurophysiological research shows that arm swing facilitates balance recovery following a perturbation and contributes to minimizing energy consumption, as well as optimizing stability and neural performance [5, 6]. Patterns of sagittal rotation of the upper limbs occur [7], with measurable EMG activity in one or more of the proximal arm muscles when walking on the treadmill, most consistently in the posterior deltoid and the triceps brachii muscles. This can be explained not by an underlying passive mechanical effect alone, but also by contributing to a possible reminiscent of quadrupedal locomotion in four-legged animals [1]. Symmetry of the movements of the extremities seems to be the initial state, whereas a disturbed symmetry may indicate a neuro-orthopedic disorder.

The effect of arm swing on gait stability is yet not fully understood. In some studies, neither significant effects of arm swing on speed nor effects on average stride time and stride time variability are found [8]. This contradicts previous conclusions from other studies, in which arm swing played a positive role in stabilizing steady-state gait. Even in healthy people, the arm swing is primarily not fully symmetrical while walking. Studies reported left-dominant arm swing groups, concluding that asymmetry was not related to handedness. This was proven in a study by Killeen et al. [4] with 334 elderly healthy adults, 91% of them being right-handers, showing that the majority presented a stronger left-arm swing. They suspected a possible connection with the fact that people also show several other movement asymmetries. For instance, when prompted to turn, children tended in 59%–79% of cases to turn counterclockwise. In addition, cultural reasons may influence arm swing behavior, which was shown in a study describing unilaterally reduced arm swing in military trained personnel [9].

Gait and arm swing evidently influence each other. To highlight one of these interdependencies, we narrowed down the field of observation and examined whether and to what extent a condition of the upper extremity exerts influence on gait patterns. The aim of this study was to find correlations between unilateral disturbed shoulder motion and gait. For this reason, several gait parameters, the DASH score, shoulder pain (NPRS), and shoulder mobility (aROM for flexion and abduction) were evaluated in patients of an orthopedic facility and in a control group of therapists.

## 2. Material and Methods

In the period from February 2018 to April 2018, 170 patients were admitted to a rehabilitation center for inpatient-rehabilitation, with 69 patients suffering from complex functional disorders of the upper extremities. Before admission, each subject was provided with comprehensive information about the study, which had been approved by the local ethics committee. 49 of them could not be included in the study due to the exclusion criteria. These criteria included restricted shoulder movement due to a bandage or cast, restriction in movement at any other joint, acute inflammatory processes, affections of the lower extremity, and neurologic and psychiatric disorders.

Two dropouts were noted because they failed to reach a walking speed of 4 km/h on the treadmill. 20 of the remaining 22 subjects participated in the study. They and a control group of ten healthy subjects underwent a gait analysis, using a Zebris gait analysis system. Eleven subjects had undergone surgery after tear of the rotator cuff, subcapital fracture of the humerus, or arthritis of the acromioclavicular joint. The other nine subjects who suffered from minor rotator cuff tears or arthritis of the acromioclavicular joint had followed a conservative therapy regimen. The shoulder side was not relevant for the admission procedure. Various gait parameters as well as the DASH score, pain at rest and in movement of the affected shoulder, and the active range of motion (aROM) for shoulder flexion and abduction were recorded.

The gender distribution in the shoulder group was 70% females and 30% males in the shoulder group and 80% females and 20% males in the control group. The study participants were on average 45.2 years old (25–70 years), with the mean age in the shoulder group of 53.8 years (39–70 years) and in the control group of 28.1 years (25–37 years). While the subjects of the two groups showed a comparable gender distribution, the mean age values between the groups were significantly different.

### 2.1. Gait Analysis

Walking can be defined as a method of locomotion involving the use of the two legs alternately to provide both support and propulsion. The challenge of walking is the abrupt transfer of body weight onto a limb that has just finished swinging forward and has an unstable alignment [7]. The terminology labelling the individual gait phases refers to the Rancho Los Amigos classification by Jacquelin Perry [10], describing here the step length and the total duration of the stance phase with its components, the loading respond, midstance, and the terminal stance phase. Gait analysis is used to describe normal gait or a disturbed gait pattern in subjects with predominantly neurological diseases or orthopedic impairments. Special analysis systems have been developed to delineate individual gait phases. Historically, a distinction was made between a swing phase and a gait phase, whereby these systems became more sophisticated and today sometimes differ in their terminology [11]. In gait analysis, the focus so far has been on the actions of the lower extremities. The contribution of the trunk and neck has also been considered, as well as of the shoulder girdle and upper extremities [12, 13]. However, a special involvement of the trunk and upper extremities [14] has been mentioned for various neurological diseases [15], of which some are still associated with their first describers, such as the neurologists Duchenne de Boulogne and Wernicke or the surgeon Friedrich Trendelenburg. Even in healthy people, the gait pattern is not constant during lifetime and changes from childhood through adulthood to senior citizens [16, 17].

### 2.2. Zebris Gait Analysis System

**Figure 1:**
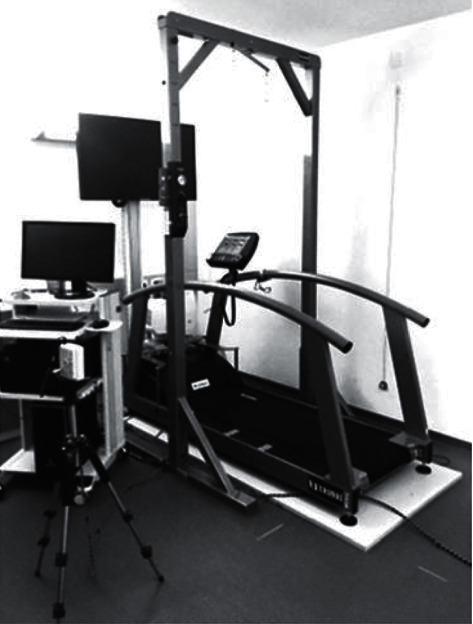
Zebris gait analysis system.

Gait analysis serves as an important tool to assess gait patterns related to functional limitations due to neurological or orthopedic conditions [18]. Treadmills with pressure transducers have been used to investigate fundamental control mechanisms in gait, disturbances associated with orthopedic or neurological disorders, and as an outcome measure to monitor the effectiveness of various clinical and neurorehabilitation trials [19]. For this study, we used a stationary treadmill (Zebris FDM-T Treadmill, Zebris Medical GmbH, Germany) (see Figure 1), which was equipped with a high frequency 120 Hz videosync system, an integrated pressure sensor mat comprising a matrix of high-quality capacitive force sensors (range 1–120 N/cm^2^, precision ± 5%), and analysis software. Approximately 10,240 miniature force sensors 0.85 × 0.85 cm are embedded underneath the belt. The high density of the sensors enables a mapping of the foot at a high resolution, which allows categorizing even slight changes in the force distribution [20].

### 2.3. Shoulder Mobility and Pain

Limited shoulder mobility and shoulder pain disturbs this interaction of the upper and lower extremities. The shoulder complex enables the elbow, forearm, wrist, and hand to be optimally positioned for activities of daily living [21]. Several musculoskeletal or neurological disorders and inflammatory diseases affect the shoulder girdle and cause pain and limited range of motion [22]. A number of tools have been designed to measure joint mobility [23], varying from simple visual estimation to high-speed cinematography or wearable inertial measurement units [24, 25]. Normal range of active movement of the shoulder has been specified by the American Academy of Orthopedic Surgeons (AAOS) to be 180°for flexion and abduction and 90°for external rotation [26]. As used in the present study, the universal full-circle goniometer is the preferred instrument for measuring the range of motion (ROM).

Shoulder pain is one of the most common musculoskeletal disorders with a lifetime prevalence estimated between 7 and 21% [27]. Chronic pain has a major impact on physical, emotional, and cognitive functions [28]. Two independent aspects of pain are commonly described: the intensity, how strong the pain feels, and the affective dimension of pain, how unpleasant the pain feels. The commonly used methods of rating pain include a visual analogue scale (VAS), verbal rating scales (VRS), and numerical pain rating scales (NPRS) [29].

### 2.4. DASH Questionnaire

Several clinical scores describe the association between improvement in pain and improvement in joint function [30]. The Disabilities of Arm, Shoulder and Hand (DASH) questionnaire was developed and validated in 1994 by the American Academy of Orthopedic Surgeons, the Council of Musculoskeletal Specialty Societies, and the Institute for Work and Health in Toronto and the Council of Musculoskeletal Specialty Societies [23, 31]. It is a patient-reported outcome measure (PROM) and has good construct validity, test-retest reliability, and responsiveness to change. This evidence has been provided for both proximal and distal disorders [32], which suggests that the DASH has a role as a measure of physical function and symptoms in any single or multiple disorders of the upper limb. The DASH is composed of 30 questions, of which 21 questions relate to physical activity, such as writing or preparing a meal, 6 questions to symptoms, and 3 questions to social role. Patients rate the symptoms or function of the upper extremity on a scale from one (no difficulty) to five (execution not possible).

### 2.5. Statistical Analysis

The data were analyzed for normal distribution with the Kolmogorov–Smirnov test, in which all but one (side difference of step length) were normally distributed (*p* > 0.05). The *t*-test for independent samples was used to determine differences between the shoulder and control group and Pearson's correlation to detect whether a pathological DASH score was related to the values of the gait analysis (step length, stance phase). The level of significance was set at *α* = 0.05. There were not any missing data. All statistical calculations were carried out using SPSS (version 27). The differences in the values (delta) were determined to present the results more clearly. The duration of the stance phase is shown in percent, with 100% describing a gait cycle (stance phase and swing phase). In a further step, we described the relations between the collected variables and the comparison between the shoulder group and the control group. The DASH score, pain at rest (NPRS) and in motion (NPRS), and aROM in flexion and abduction were the independent variables.

## 3. Results

As shown in Table 1, we recorded intragroup and intergroup differences in the duration of particular elements of the stance phase. The inter-group differences were significant for all elements of the stance phase (loading response: *p* = 0.021; midstance: *p* = 0.033; and terminal stance: *p* = 0.019). Small differences were found for the duration of the total stance phase. In the shoulder group, the mean value of the duration of the total stance phase on the affected side took 63.45% ± 0.54 of the gait cycle and on the unaffected side 63.45% ± 1.11. These differences were comparable to those in the control group (63.21% ± 1.13% on the left side and 63.254% ± 0.81 on the right side).

In the shoulder group, the intragroup difference of the step length between the affected and the unaffected sides (1.45 cm ± 1.10) was not significant (*p* = 0.54). Comparing the affected side to the unaffected side, we found in the shoulder group different intragroup results for the duration of the loading response (shortened by 1.03% ± 0.75), the midstance (shortened by 1.07% ± 0.66%), and the terminal stance phase (prolonged by 1.03% ± 0.73%).

We recorded significant intergroup differences for active shoulder flexion of the affected side (117 ± 23.81 vs. 177 ± 4.0 degrees) and abduction (92.75 ± 24.73 vs. 177.5 ± 2.5 degrees). In addition, intragroup difference for flexion and abduction was recorded, which were worse in the shoulder group.

The average values of the DASH-questionnaire ranged from 37.47 in the shoulder group to 24.17 in the control group. We could not quantify any shoulder pain in the control group, while in the shoulder group the NPRS for pain at rest was 2.75 ± 2.31 and for pain in movement was 5.30 ± 2.32.

**Figure 2:**
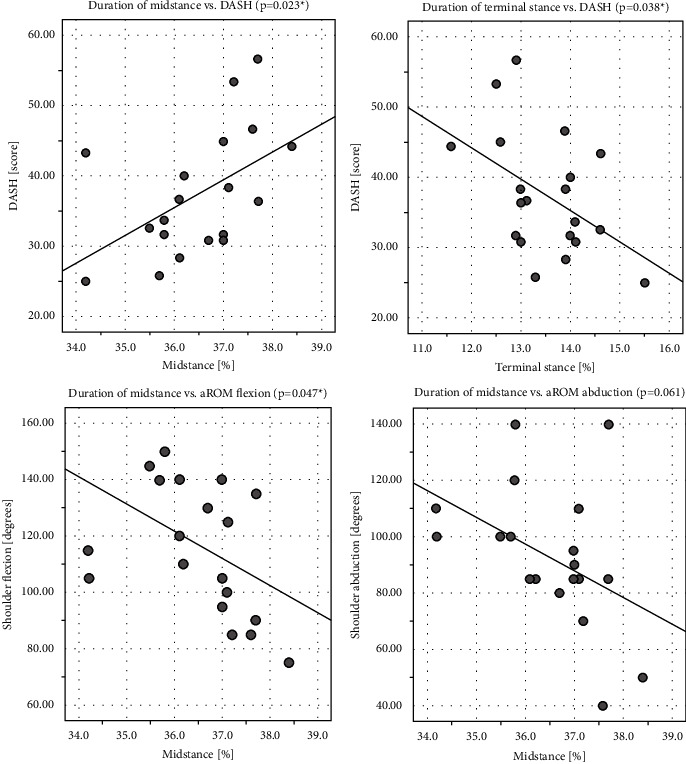
Shoulder group: correlation determination between DASH and duration of the midstance and the terminal stance phase and duration of the midstance phase and the aROM of shoulder flexion and abduction (^*∗*^asterisks indicate significance). (a) Duration of midstance versus DASH (*p* = 0.023^*∗*^). (b) Duration of terminal stance versus DASH (*p* = 0.038^*∗*^). (c) Duration of midstance versus aROM flexion (*p* **=** 0.047^*∗*^). (d) Duration of midstance versus aROM abduction (*p* **=** 0.061).

In the shoulder group, we found significant correlations between the DASH and the midstance phase (correlation coefficient *r* = 0.51, *p* = 0.023) and the terminal stance phase (correlation coefficient *r* = –0.47, *p* = 0.038). This also applies for the results of shoulder mobility and the midstance phase (Figures 2(a)–2(d)). There was a significant correlation between the duration of the midstance phase and shoulder flexion (*r* = –0.45, *p* = 0.047), and a moderate correlation between the midstance phase and shoulder abduction (*r* = –0.43, *p* = 0.061).

The duration of the midstance phase was prolonged in patients with limited shoulder flexion and abduction and elevated DASH score. The duration of the terminal stance phase was influenced by the DASH results with a prolongation of the terminal stance phase in patients with a lower DASH score. All other correlation calculations showed no significance (Table 2). Shoulder pain, restricted mobility, or a higher DASH score showed no significant correlation with step length or duration of the total stance phase, whereas limited shoulder flexion is associated with restricted arm swing, which in turn affected the duration of the midstance phase.

## 4. Discussion

As was expected in an orthopedic rehabilitation clinic, we were not able to recruit a comparable control group of unimpaired subjects. Therefore, the sample of the control group consisted of members of the rehabilitation team with a mean age of 28.1 years compared to 53.8 years in the shoulder group. It is well described in the literature that age-associated changes in gait parameters affect, amongst other parameters, the symmetry, speed, and length of step [33]. In order to minimize this effect, patients with conditions of the lower extremity were excluded. In addition, we set a threshold of at least 4 km/h walking speed that had to be reached.

Kahn et al. [34] expected subjects with acquired brain injury (ABI) to suffer from abnormal upper limb kinematics resulting in a negative impact on gait, balance, dynamic upper limb function, and activities of daily living. In a study of 42 subjects with ABI, they identified significantly more shoulder abduction and elbow flexion while walking, with approximately half the cohort exhibiting a more fixed elbow flexion pattern throughout. Other members of the cohort showed excessive movement in and out of elbow flexion and increased shoulder abduction variability. Apart from that, Malawade et al. [35] figured out the impact of limited arm swing on gait in a study with 20 healthy children (6 to 12 years). For this reason, they recorded the average values of step length, stride length, and step width with normal arm swing, dominant arm bound and with both arms bound. The average stride length (75.1 cm) decreased to 62.0 cm with the dominant arm bound, while the average stride length was barely affected when the child could not move both arms. They concluded that the unilateral inhibition of arm swing had a significant effect on the step length, stride length, and step width of children in the age group of 6 to 12 years. Their results did not correlate with the findings of the present study, in which shoulder disturbances only had a minor effect on the step length.

In a study on 25 subjects after unilateral transhumeral amputation and prosthetic fitting, Topuz et al. [36] recorded differences in arm swing and spatiotemporal characteristics of gait compared to the control group. They found that step and stride lengths were shorter and gait velocity and cadence were lower in the amputee group compared to the healthy group. Their findings did not match our results, in which the total step length was virtually not influenced by reduced shoulder mobility. Evidently, the impact of upper limb restrictions on gait depends on the extent of the restriction or deformation of the upper limb. Our results indicate that if upper limb symmetry is not affected, a partial restricted shoulder movement has an impact on particular elements of the stance phase, but only barely on step length or duration.

In contrast to our results, shoulder immobilization does not necessarily lead to worse gait patterns. Lin and colleagues showed in their meta-analysis that arm sling use improved gait by significantly increasing walking speed in patients with poststroke hemiplegia [37]. They explain this effect with substantial upper limb weakness and vertical glenohumeral subluxation, eventually resulting in hemiplegic shoulder pain. On the other hand, their results are not in accordance with those in a systematic review by van Bladel et al., who found no strong evidence regarding a potential benefit of wearing an arm sling on balance and gait for stroke patients [38]. Diverging data and results reveal that further studies are required to explain the relationship between the movements of the upper and lower extremities.

### 4.1. Strength and Weakness of the Study

The study highlights the effects of disturbed shoulder movement on gait at a certain walking pace, but does not provide information about the results at different speeds. In addition, walking patterns on a treadmill differ from real-life situations. The subjects suffered from various complaints of the shoulder girdle. Thus, the informative value is reduced due to the small number of participants and the inhomogeneity of shoulder affections. In contrast to the technical three-dimensional representation of the shoulder movement using electronic devices, only the ROM was measured in our study, which can lead to distortion of the results. As mentioned above, the mean age values between the groups was significantly different; therefore, possible bias effects cannot be ruled out. We recommend further studies with larger and more homogenous samples for both the shoulder and the controls.

## 5. Conclusion

Gait analysis systems are efficient tools for objectively identifying and evaluating asymmetries when walking. In general, normal arm swing behavior is related to a normal gait pattern. Abnormal arm swing can result in abnormal gait, with a partially restricted shoulder movement having an impact on particular step characteristics. The results of the present study reveal the influence of limited shoulder function and a moderate unilateral restriction of shoulder mobility on the duration of elements of the ipsilateral stance phase. We found a significant impact of the DASH score and shoulder flexion on the duration of the midstance phase, and the DASH score on the terminal stance phase. We conclude that impaired shoulder function and limited arm swing exert influence on elements of the stance phase in human gait.

## Figures and Tables

**Table 1:** Gait parameters of shoulder group compared to control group.

Shoulder group (*n* = 20)	Affected side (duration of gait cycle in percent)	Unaffected side (duration of gait cycle in percent)	Mean value and SD of side differences (affected-unaffected side)

Total stance phase	63.45% ± 0.52	63.45% ± 1.11	1.04% ± 0.67
Loading response	13.37% ± 0.90	13.63% ± 0.94	1.03% ± 0.75
Midstance	36.50% ± 1.07	36.55% ± 0.57	1.07% ± 0.66
Terminal stance	13.52% ± 0.89	13.38% ± 0.87	1.03% ± 0.73
Step length	57.20 cm ± 3.25	57.25 cm ± 3.49	1.45 cm ± 1.10

Control group (n **=** 10)	Left side	Right side	Mean value and SD of side differences (left-right side)

Total stance phase	63.21% ± 1.13	63.25% ± 0.81%	0.54% ± 0.53
Loading response	13.17% ± 0.96	13.27% ± 0.99%	0.43% ± 0.27
Midstance	36.75% ± 0.85%	36.78% ± 1.16	0.53% ± 0.51
Terminal stance	13.28% ± 1.0%	13.19% ± 0.97	0.43% ± 0.28
Step length	61.0 cm ± 3.23	61.6 cm ± 2.71	1.20 cm ± 0.91

**Table 2:** Cross tabulation of gait and secondary outcome parameters.

		Step length	Total stance phase	Loading response	Midstance	Terminal stance
DASH	Significance *p* (correlation)	0.303 (0.243)	0.988 (−0.004)	0.438 (−0.184)	0.023^*∗*^ (0.507)	0.038^*∗*^ (−0.468)
NPRS at rest		0.359 (−0.217)	0.268 (−0.260)	0.165 (−0.323)	0.653 (0.107)	0.863 (0.041)
NPRS in movement		0.766 (−0.071)	0.679 (−0.099)	0.322 (−0.233)	0.704 (−0.091)	0.182 (0.311)
aROM flexion		0.654 (0.107)	0.913 (0.026)	0.296 (0.246)	0.047^*∗*^ (−0.449)	0.159 (0.328)
aROM abduction		0.883 (0.035)	0.355 (0.218)	0.106 (0.373)	0.061 (−0.426)	0.212 (0.292)

^*∗*^Asterisks indicate significance; correlation coefficients are shown in parenthesis.

## Data Availability

The data of this study can be made available by the corresponding author upon request.
